# Immunophenotype of Normal and Myelomatous Plasma-Cell Subsets

**DOI:** 10.3389/fimmu.2014.00137

**Published:** 2014-03-31

**Authors:** Nelly Robillard, Soraya Wuillème, Philippe Moreau, Marie C. Béné

**Affiliations:** ^1^Service d’Hématologie Biologique, Laboratoire de Biologie, CHU de Nantes, Nantes, France; ^2^Service d’Hématologie Clinique, CHU de Nantes, Nantes, France

**Keywords:** plasma cells, multiple myeloma, flow cytometry, bone marrow, immunophenotype

## Abstract

Plasma cells (PCs) are essentially characterized by the co-expression of CD138 and CD38, which allows their identification in flow cytometry in bone marrow (BM), peripheral blood, or cell suspensions from tissues. These terminally differentiated B-cells may lose the expression of surface CD19 and that of CD20 while retaining CD27. When malignant, they can gain a number of other markers such as CD28, CD33, CD56, or CD117 and lose CD27. Moreover, since each PC is only able to produce a single type of immunoglobulins (Igs), they display isotypic restriction and clonal malignant PCs can be further characterized by their homogeneous expression of either kappa or lambda light chains. In multiple myeloma (MM), such PC clones produce the Ig identified in plasma as an abnormal peak. In the BM where they essentially accumulate, these PCs may however display various immunophenotypes. The latter were explored in a two-way approach. Firstly, the various subsets delineated by the selective or common expression of CD19 together with combined CD56/CD28 were explored in normal and MM BM. Then, other aberrant markers’ expression was investigated, i.e., CD20, CD27, CD33, CD56, CD117. These data were compared to literature information. They underline the vast heterogeneity of MM PCs possibly accounting for the various answers to therapy of MM patients.

## Introduction

Plasma cells (PC) represent the terminal differentiation stage of mature effector B-cells. They result from the clonal proliferation of an activated B-cell after antigen recognition, yielding both immunoglobulin (Ig)-producing PC and memory B-cells increasing the individual’s ability to generate a humoral immune response upon further encounter of the same antigen. In multiple myeloma (MM), such a PC clone becomes dysregulated and keeps proliferating and secreting Igs, identifiable in the patient’s serum as a monoclonal Ig peak. Regular measurements of this peak level allow to appreciate patients’ response to therapy. An assessment of the bone marrow (BM) infiltration by malignant PC provides complementary information at diagnosis, in evaluating the importance of the clone. Such an evaluation is especially useful during follow-up and therapy if the serum Ig peak disappears, i.e., allowing to characterize minimal residual disease (MRD) (1). PCs are first identified morphologically on BM smears. Flow cytometry (FCM) is a valuable addition to this observation, allowing to differentially appreciate the subsets of coexisting normal and MM PC. FCM relies on an estimation of the restricted usage of Ig light chains by the clonal population, usefully completed by an assessment of other immunophenotypic features of PC. Choice of the latter, however, is complicated by the diversity of aberrant markers reported to be expressed on clones and subclones of MM PC. Quite a number of studies in the literature have reported on the immunophenotype of both normal and MM PCs (2–5). The most salient feature of normal PC, compared to mature B-cells, is the co-expression of CD138 and CD38, which allows their identification in FCM in BM, peripheral blood (PB), or cell suspensions from tissues (6). These terminally differentiated B-cells may lose surface expression of the pan-B marker CD19 and stop expressing CD20 while retaining the memory-associated antigen CD27. Identification of intracytoplasmic light chains allows to appreciate the polyclonal nature of BM-infiltrating normal PC, which use kappa and lambda light chains at a ratio comprised between 0.76 and 2.21 [(7) and personal data].

When malignant, PC can gain a number of other markers such as CD28, CD33, CD56, or CD117 and lose the memory-associated antigen CD27. However, since each PC is only able to produce a single type of Igs, they display isotypic restriction and clonal malignant PCs can be further characterized by their homogeneous expression of either kappa or lambda light chains.

In 2011, Peceliunas et al. (8) reported on the characterization of different subsets among normal PC, using a two-tubes, six-colors approach combining the investigation of surface CD45, CD19, CD38, CD138, CD20, and CD56. This allowed identifying four different PC subsets based on the expression or not of CD19 and CD56. The latter were dubbed “normal” PC when expressing CD19 in the absence of CD56, “aberrant” when lacking CD19 yet expressing CD56, and respectively “double positive” and “double negative” for the other two. In a series of 11 normal BM samples, all subsets were observed in 5 subjects, 5 were lacking “double positive” PC (median for positive cases, 3%), and 1 was lacking a significant amount of “aberrant” PC (threshold 10^−3^). The subset of “normal” CD19+/CD56− PC represented between 37 and 72% of BM PC. The median proportions of aberrant and double negative PC were respectively 10 and 30%. These data obtained in normal BM were compared to those seen in 27 BM samples from relapsed/refractory MM patients. The authors concluded that the presence of these various subsets in normal PC limited the interest of FCM for the identification of MM PC. Of note, light-chain restriction was not investigated by these authors.

We developed along the same lines an FCM strategy separating BM PC in four subgroups, based on the co-expression or not of surface CD19 and of CD28/CD56. In addition, however, light-chain usage restriction was investigated in each of these compartments, allowing to accurately segregate normal and malignant PC. We further explored PC in MM patients by assessing the presence, absence, or partial expression of other differentiation antigens. Data reported here highlight the great heterogeneity of MM PC, as identified by FCM, indicating that subclones, detectable at diagnosis, may drive the evolution of this disease in spite of hopefully efficient therapies.

## Materials and Methods

A total of 139 samples from MM patients (138 BM and 1 CSF) at diagnosis were stained for FCM after Ficoll density gradient separation (3 samples) or red cell lysis (Versalyse, Beckman Coulter, Miami, FL, USA), then permeabilized (Intraprep, Dako, Glostrup, Denmark) for the detection of intracytoplasmic light-chains usage (7). As shown in Table 1, in a first tube, the seven-color combination CD45 (to gate leukocytes), CD19, CD38, CD138, CD28+CD56, for surface staining, completed by intracytoplasmic investigation of kappa and lambda chains was used to explore the four different subsets combining the use of CD19 and CD56+CD28. These subsets were selectively examined among the whole population of CD38+/CD138+ PC. In 132 of these samples, PC expression of CD28 and CD56 was also investigated separately. Additionally, several other PC surface markers were assessed individually in the other combinations shown in Table 1: CD20 (*n* = 130), CD27 (*n* = 124), CD33 (*n* = 131), and CD117 (*n* = 130). A series of 26 normal BM samples from BM donors or hospitalized patients without hematological malignancy (mostly ITP) was studied with the same seven-color combination of tube 1.

**Table 1:** **Panels of antibodies used for immunophenotyping of MM PC**.

	FITC	PE	PC5	PC7	APC	APC H7	V450
Tube 1	Lambda	CD56+CD28	CD138	CD19	Kappa	CD45	CD38
Tube 2	CD14	CD56	CD138	CD19	CD33	CD45	CD38
Tube 3	CD20	CD22	CD138	Control	CD19	CD45	CD38
Tube 4	Control	CD28	CD138	CD19	CD117	Control	CD38
Tube 4	CD45	Control	CD138	CD27	Control	Control	CD38

All samples were incubated using at least 10^6^ nucleated cells. Labelings were analyzed on FACSCanto II flow cytometers (BD Biosciences, San Jose, CA, USA) using the Diva (BD Biosciences) software as reported elsewhere (7). Statistical analyses (medians, ranges, partition, graphs) were performed using MedCalc (Marienkirche, Belgium).

## Results

In MM patients, light-chain restricted PC represented a median of 97% of total CD38+/CD138+ PC, levels ranging between 76.5 and 100%. Kappa chains were used predominantly (93 cases, 67%). PCs were detected in all MM samples (median 7.5% of nucleated cells, range 0.04–91) and all normal BM samples (median 0.29% of NC, range 0.037–1.2).

**Figure 1:**
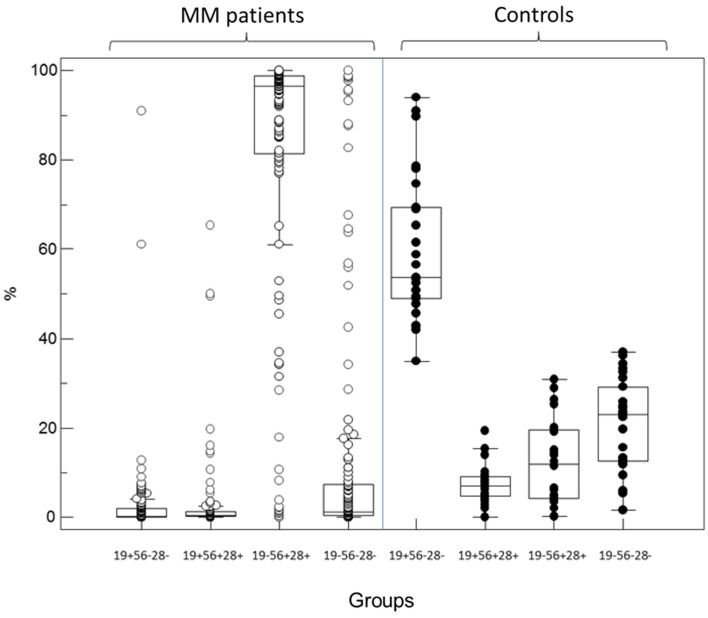
**Partition of PC percentages among the four immunophenotypic subgroups defined by the expression or not of CD19 and the combination CD56/CD28 in MM patients (left, open circles) and controls (right, black circles)**. In diagnosis samples from MM patients, most of the abnormal plasma cells belong to the CD19− CD56+/28+ subgroup 3 while most but not all normal PC in controls retain expression of CD19 in the absence of CD56 and CD28 (subgroup 1).

Independently of light-chain restriction, the four expression patterns of CD19 and CD56/CD28 were identified as groups (1) CD19+/(CD56/CD28)−, (2) CD19+/(CD56/CD28)+, (3) CD19−/(CD56/CD28)+, and (4) CD19−/(CD56/CD28)−. As shown in Figure 1, the predominant immunophenotypic population in MM samples was that of PC lacking CD19 and expressing either CD56 or CD28 (group 3). Medians were respectively 0.3, 0.5, 96.5, and 1.2% of all PC for the four subsets. Of note, PCs with a “normal” immunophenotype (group 1) were at very low levels, with a maximal value of 12.8% except in two patients for whom this was the major monoclonal population with light-chain usage restriction. Light-chain usage was polyclonal in the other samples of this group, as these cells represented the normal component of BM PC, besides the MM population. Only one subset was seen in 50 samples (group 1, *n* = 1, group 3, *n* = 48, group 4, *n* = 1). A combination of groups 2 and 3 was observed in 23 cases, of groups 3 and 4 in 28, and of the three groups 2, 3, and 4 in 32. Finally, in six cases, light-chain restricted cells were seen in all four possible subsets (1, 2, 3, and 4). The size of clonal subsets in one of the groups could be as small as 0.1% of the PC.

**Figure 2:**
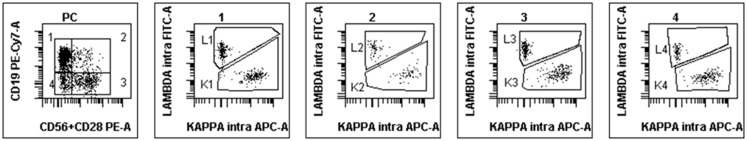
**Example of FCM scattergrams of a control BM sample**. The left panel shows the partition in four subgroups of CD38+/CD138+ cells (initial gate, not shown) according to the expression or not of CD19 and the combination of CD56/CD28. Most of normal PC are in subgroup 1 but cells can also be seen in the other compartments. Polyclonality is confirmed by the investigation of cytoplasmic light chains in the four subsequent scattergrams gated on groups 1–4 from left to right.

**Figure 3:**
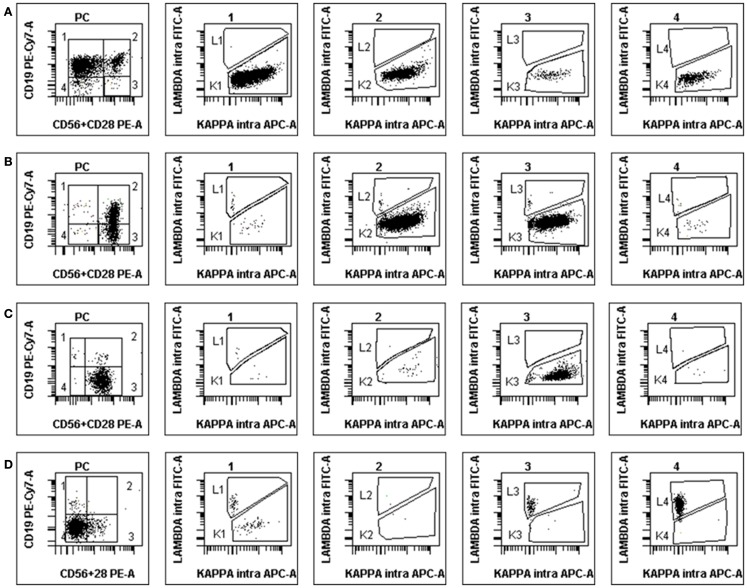
**Examples of abnormal PCs in MM BM samples at diagnosis**. The disposition is the same as in Figure 2, light-chain expression being shown in the four subsets (1–4). **(A)** Patient with κ-restricted abnormal PC presents mostly in subgroups 1 and 2 but also 3 and 4. **(B)** Patient with κ-restricted abnormal PC presents mostly in subgroups 2 and 3, with polyclonal residual normal PC in subgroup 1. **(C)** “Classical” MM patient with the vast majority of κ-restricted abnormal PC present in subgroup 3, i.e., lacking CD19 and expressing either CD56 or CD28 (or both). **(D)** Atypical MM patient with abnormal PC most of which lack all three surface antigens yet clearly display λ-restriction in subsets 4 and 3. Note the small population of residual polyclonal normal PC in subgroup 1.

By contrast, in normal BM samples, a median of 53.7% of PC was found to retain CD19 expression alone (group 1) and 7% expressed CD19 together with CD28/CD56 (group 2). However, 12% expressed one or both of the latter markers in the absence of CD19 (group 3) and 23% were completely negative for these surface antigens (group 4). Each of these four populations was however clearly polyclonal and displayed a normal partition of kappa and lambda light-chains usage, with a median K/L ratio of 1.27. Figure 2 displays the polyclonal subsets of normal BM PCs in the four FCM compartments of control BM. By contrast; Figure 3 shows how the light-chain restricted MM PCs could be retrieved in the various compartments.

**Figure 4:**
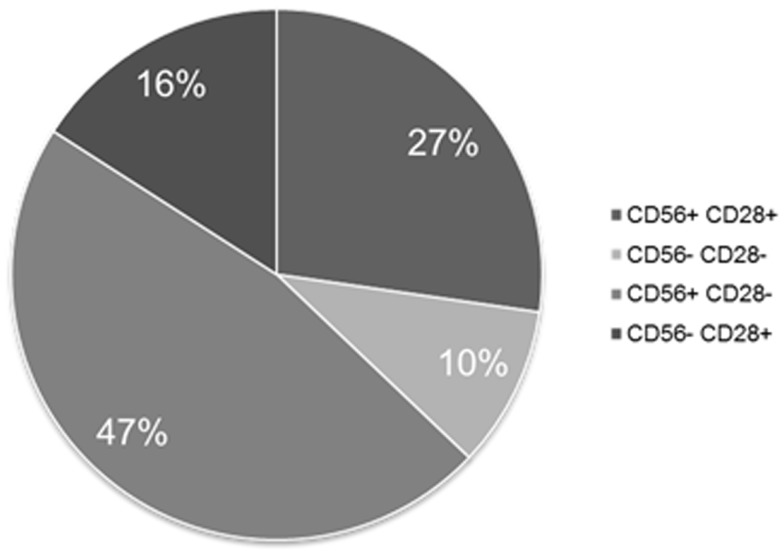
**Expression patterns of CD56 and CD28 on MM PCs when tested separately**. This figure shows that 90% of MM patients express at least one of these two aberrant markers.

Separate exploration of MM PC retrieved CD56 expression in 74% of the cases and of CD28 in 40%. Figure 4 shows the partition of the various co-expression patterns observed for these two differentiation antigens on MM PC, the most frequent being CD56^+^CD28^−^.

**Figure 5:**
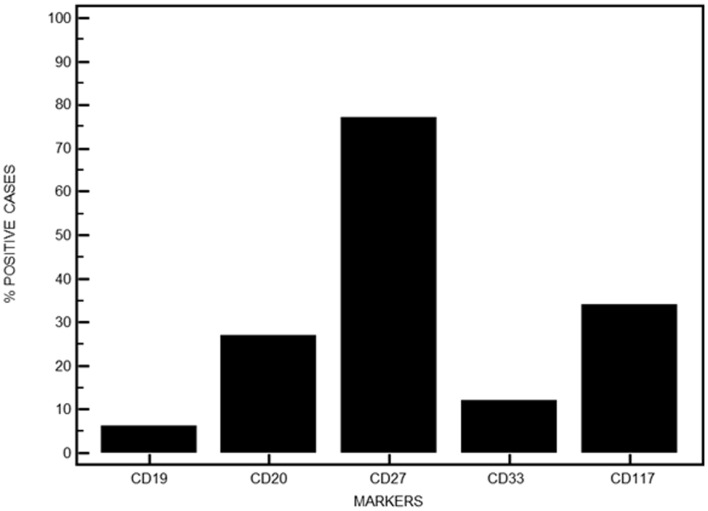
**Immunophenotypic features of MM PC when investigating other aberrant surface markers**. Data are expressed in percentages for the series presented here.

The expression of CD19 was retained on MM PC in eight patients with high levels above 50% in only five. CD20 expression was absent in 73% of the cases. Conversely, expression of CD27 was retained in 77 patients while respectively 16 and 45 cases expressed CD33 or CD117 (Figure 5). Table 2 displays the heterogeneity of immunophenotypic patterns observed in this series of MM PC. Although most samples showed a homogeneous absence or presence of differentiation antigens expression on PC, quite a number showed intermediate levels indicative of putative subclones. Only follow-up would have allowed to see whether any of these subclones ultimately emerged as therapy-resistant, but this heterogeneity highlights the complexity of MM pathophysiology.

**Table 2:** **Expression of aberrant markers on PC from MM patients at diagnosis**.

	No expression (*n*)	Expression on	Intermediate expression
		all PC (*n*)	(*n* = % expression range)
CD20	95	22	13 (15–84)
CD27	47	47	30 (12–88)
CD33	114	4	12 (7.5–86)
CD117	86	26	19 (12–91)

Finally, a single weak (*r* = 0.16) negative correlation was observed between CD27 and CD117 expression when comparing differentiation antigens expression.

## Discussion

Morphological observation of PC, either the rare normal PC or sheets of abnormal MM PC on BM smears or biopsies, already discloses heterogeneity in the shape and staining of these cells. Although this could be due to the level of differentiation and secreting activity of PC (9), it could have been assumed that the clonal population producing a narrow peak of Ig in MM would be quite homogeneous. The only real level of homogeneity observed is in fact in the consistent isotype restriction of the monoclonal Ig. At first glance, the heavy involvement of patients’ BM by light-chain restricted PC (median 97%) could indeed suggest a homogeneous population. However, other immunophenotypic features are quite variant within the clone, possibly denoting the presence of subclones that retained the initial rearrangement of Ig genes, but differ in other molecular features. Several data have underlined the genomic heterogeneity of MM PC (10, 11), yet this has not been straightforwardly correlated to that of their immunophenotype. Indeed, the work of Peceliunas et al. (8) described in the introductive part of this manuscript is one of the first systematic attempts at dissecting the various populations present within a rather easily defined cluster of CD38+CD138+ PC. Here, we demonstrate in a large series of MM patients at diagnosis that this heterogeneity is retrieved by the concomitant presence of PC with the same light-chain restriction in at least two and up to the four subsets defined by the CD19−CD28/CD56 combination in 64% of the patients. Moreover, even in the 50 cases where PC belonged to only one of these four groups, great heterogeneity in the other immunophenotypic markers studied was observed. This is especially true for the markers with “partial” expression, denoting at minimal a positive and a negative subclone for the marker of interest.

One striking feature of MM PCs is their wide heterogeneity in CD45 expression, which greatly underscores the normally useful backgating of hematopoietic subsets on a CD45/SSC “cartography” (12). This has been reported previously (13, 14) and is at clear variance with the classical very homogeneous bright CD45 staining of low SSC lymphocytes (12).

Other abnormal expressions of differentiation markers have been reported on PCs, but more as being useful to discriminate between normal and MM PC than by being representative of clonal heterogeneity (14). Liu et al. (14) explored the features of normal PC and reported on heterogeneous expression of CD45, high but also heterogeneous (range 52–97%) expression of CD19 and loss of CD20. They found that MM PC expressed more aberrancies than normal PC, usually with a stronger expression of such markers as CD28, CD56, or CD117. In this study, they also insisted on the importance of light-chain restriction usage determination to discriminate between polymorphic and MM PC. The review by Kumar et al. (15) and work from Paiva et al. (16) summarize the abnormalities to be expected on MM PC with regard to the major antigens explored here, i.e., CD20, CD27, CD28, CD56, CD33, CD117, with the addition of CD81, positive on normal PC but lost on MM PC. Of note, the ~30% expression of CD117 observed in our series is similar to previously reported data (17–19).

The impact of immunophenotypic variability on prognosis was hinted in 2008 by Mateo et al. (20). These authors identified three risk categories: poor risk (CD28+ CD117−), intermediate (either both markers negative or both positive), and good risk (CD28− CD117+). Here, we observed these four types of immunophenotypes, but without any correlation between CD28 and CD117 expression. Surprisingly, we observed however a negative correlation between the good prognosis markers CD117 and CD27 (21, 22). This might suggest that both these markers should be investigated at diagnosis to assess the possible evolution of the patients, and that those negative for both markers (23% in this series) might deserve special medical attention.

The retained expression of CD20, observed here in 27% of the patients is in line with previous reports (23). That this subset of patients could be accessible to therapy with anti-CD20 monoclonal antibodies has however not been confirmed so far (24).

Finally, CD33 expression, which could also potentially be accessible to immunotherapy, is in this series much lower than in an earlier paper from our group (25).

The persistence or disappearance of B-lineage or co-stimulation markers on MM PC can be understood as an evolution of the malignant cells within the B-lineage and could be a reflect of molecular events that occurred in the initial diseased B-lymphocyte. The presence of such more myeloid-restricted antigens as CD117 or CD33 could suggest anomalies developed earlier in the differentiation pattern of the malignant progenitor. CD56 expression, because of its wide promiscuity and major role as adhesion molecule, could be more related to events allowing for MM PC dissemination and contacts with the environment within the BM or in extramedullar localizations. None of these hypotheses has however been verified.

In conclusion, this work provides further information on the intrinsic heterogeneity of normal PC, retained and somewhat exacerbated in MM PC. The most obvious difference is the predominance of CD19+ CD28/56+ in normal PC, a subset clearly minor in MM patients. The most stable feature remains the restricted usage of the light chain rearranged in the initial cell, which can also be found in any of the four subsets delineated by CD19−CD28/56, as well as associated with variable levels of other differentiation antigens. Better definition of an individual patient at diagnosis might help to better track therapy-resistant subsets. Although this has not been fully investigated yet, it might appear that specific immunophenotypic patterns could be associated with a better sensitivity to specific drugs or drugs combinations.

## Conflict of Interest Statement

The authors declare that the research was conducted in the absence of any commercial or financial relationships that could be construed as a potential conflict of interest.
